# *In Vivo* Imaging of Neutrophil Extracellular Traps (NETs): Visualization Methods and Outcomes

**DOI:** 10.1155/2020/4192745

**Published:** 2020-02-01

**Authors:** Sultan Z. Alasmari

**Affiliations:** Clinical Laboratory Sciences, Faculty of Applied Medical Sciences, King Khalid University, Abha 62529, Asir Region, Saudi Arabia

## Abstract

Neutrophils comprise the first line of innate immune defense during a host-pathogen interaction. They attack microorganisms directly through three different methods, of which, phagocytosis and degranulation have been known and well-studied for decades. The formation of neutrophil extracellular traps (NETs) is the third and unique method, which was unveiled in 2004. Since then, many studies on NETs have been carried out. However, only few have successfully demonstrated the activity of NETs *in vivo*. Results of the *in vivo* studies on NETs have strengthened our understanding of their role in different situations. This review highlights the main *in vivo* studies, which have contributed in extending our understanding of the role of NETs during infections and diseases, thus indicating their advantages and limitations.

## 1. Introduction

Neutrophils are known to play a crucial role in immune defense. They attack microorganisms via three distinct methods, which include phagocytosis, degranulation, and the formation of neutrophil extracellular traps (NETs) [1]. Phagocytosis and degranulation have been known and well-studied for decades. However, questions still exist about the formation of NETs, which were first described in 2004 by Brinkmann et al. [2]. They demonstrated that stimulated neutrophils undergo unusual morphological changes and produce web-like structures termed NETs, which were composed of DNA, histones, and granular proteins [2]. These structures trap and kill the invading pathogens extracellularly. The formation of NETs is stimulated through the activity of chemicals such as phorbol-12-myristate-13-acetate (PAS) and calcium ionophore [2–4]. It has been reported that the formation of NETs is induced by the presence of several pathogens including Gram-positive bacteria such as *Staphylococcus aureus* [5] and *Streptococcus pyogenes* [6]; Gram-negative bacteria such as *Escherichia coli* [7], *Shigella flexneri* [2], *Yersinia enterocolitica* [8], and *Yersinia pseudotuberculosis* [8]; viruses such as HIV-1 [9]; and other organisms [10].

Furthermore, it has been determined that NETs are involved in a variety of conditions such as cancers and vascular diseases like atherosclerosis, small vessel vasculitis (SVV), and thrombosis [11–14]. The majority of the studies on NET formation have been carried out *in vitro*. However, in few studies, the formation of NETs in living organisms has been microscopically analyzed. This review focuses on the main *in vivo* studies, which have been conducted to determine the role of NETs in infections and diseases, thus indicating their advantages and limitations.

## 2. The Morphology and Mechanisms of NETs

NETs are fragile fabrics composed of nuclear components and granules, which trap and, in many cases, kill pathogens extracellularly. High-resolution scanning electron microscopy (SEM) has revealed that this fabric (NETs) is composed of smooth stretches and globular domains aggregating into large threads [2]. The use of immunofluorescence staining methods has revealed that NETs consist of DNA, histones, and primary granule proteins such as neutrophil elastase (NE), myeloperoxidase (MPO), and cathepsin G. Lactoferrin and gelatinase are the secondary and tertiary granular portions contained within NETs, respectively [2]. To date, three distinct forms of NET release have been identified.

**Figure 1:**
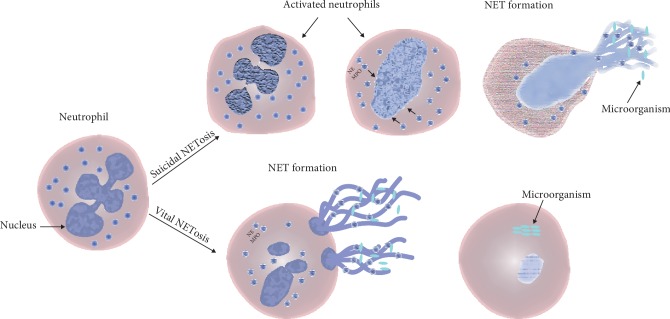
Mechanisms of NET formation. Upon activation, phenomenal morphological changes are observed during suicidal NETosis, which is followed by cell rupture and subsequent cell death. Contrarily, in vital NETosis, NETs are released rapidly by the active and functional NET-releasing neutrophils through blebbing of the nuclear envelope and vesicular exportation.

The first novel mechanism of NET formation involves the occurrence of morphological changes in activated neutrophils. Activated neutrophils tend to flatten and lose the lobules of their nuclei, after which, the chromatin is decondensed, followed lastly by a nuclear detachment of the inner and outer membranes. Besides, the separation of the granules is also observed. After 1 h of activation, the nuclear envelope breaks into pieces. Finally, the cells round up until the cell membrane ruptures and ejects their internal contents into the extracellular space forming NETs [2]. This type of NET formation is known as a suicidal NET or NETosis (Figure 1). The term NETosis was first coined by Steinberg and Grinstein to describe suicidal NETosis [15]. The second form of NET release is termed as vital NETosis, during which stimulated neutrophils remain active and functional following the NET release (Figure 1) [16, 17]. The major difference between suicidal and vital NETosis is that the suicidal NET release occurs slowly whereas vital NETosis occurs rapidly [17, 18]. It has been observed that vital NETosis is induced following bacterial infections while chemical stimuli such as phorbol-12-myristate 13-acetate (PMA) usually induce suicidal NETosis [17]. The mechanisms for NET release are different for suicidal and vital NETosis (Figure 1). Neutrophils stimulated using PMA, uric acid crystals, or *Candida albicans* induce NET release depending on the induction of NADPH oxidase and activities of elastase and MPO [2, 5, 19–21].

However, bacteria and their products have been found to induce NET release through mechanisms involving toll-like receptors (TLRs) and integrins [16–18, 21, 22]. The third form of NET release is observed upon the activation of neutrophils due to saliva. Neutrophils elicited through the saliva undergo NETosis, which is independent of the activities of NADPH oxidase, elastase, and integrins [21]. Additionally, saliva-induced NETs are resistant to the effects of DNase and have higher bactericidal activities [21]. The second and third types of NETosis are observed *in vivo* and discussed in detail below.

## 3. *In Vivo* Methods for Visualization and Quantification of NETs

NET release was first visualized *in vitro*. The first *in vitro* study reported that neutrophil lysis results in the formation of fragile web-like structures known as NETs [2]. This discovery has led researchers to carry out extensive studies on the role of NETs in countering infection and during inflammation. The majority of the studies involving NETs have been conducted *in vitro*. However, a few reports have presented the effect of NET release *in vivo*. Visualization of NETs *in vitro* is dependent on the composition of their histones, MPO, neutrophil elastase, and DNA. Despite visualizing NETs successfully *in vitro*, questions on their effects *in vivo* have remained unclear due to the limited number of *in vivo* studies. Most of the articles published to date on *in vivo* studies involving NET quantification have focused on their direct visualization using advanced microscopic techniques such as spinning-disk confocal intravital [16, 23–25] or two-photon microscopy [26, 27].

Fluorescent labeling of microorganisms is another method used for the direct visualization of pathogen interaction with NET-releasing neutrophils and NET *in vivo* [16, 23]. NETs have been directly visualized *in vivo* using several methods such as staining of the extracellular DNA using membrane-impermeable DNA dyes [16, 23–28] and fluorescently labeled antibodies specific to the NET components such as histone and neutrophils elastase [16, 23–25, 27]. Additionally, cell-permeable and cell-impermeable DNA dyes have been used in combination in some studies to distinguish between the intercellular and extracellular DNA of the cells [16]. Cell-permeable DNA dyes have been administrated to enable nuclear imaging [16]. To investigate the interaction with the presence or absence of NETs *in vivo*, another method involving the use of DNase has been employed, which has verified that NETs are composed of DNA and also enabled evaluation of its degradation [23–25, 27]. Some studies have used an inhibitor of neutrophil elastase to demonstrate the degradation of NET [24, 25]. Neutrophil depletion *in vivo* has been employed to determine the importance of neutrophils as a source of NET through treatment using neutrophil-specific antibodies [23–25]. NETs have been quantified by determining their sizes using a velocity imaging software [16] and the ImageJ software [23, 24]. NETs were quantified *in vivo* by counting their numbers in 10 randomly selected fields of intravascular spaces, hepatic sinusoids, and pulmonary capillaries using intravital multiphoton microscopy (MPM) imaging. The total number of NETs in these 10 fields was summed and expressed as the number of NETs present per field of view [27]. The behaviors of neutrophils were examined by tracking an individual neutrophil, and its velocity, distance, and displacement were determined using the Volocity software [16]. Interestingly, all *in vivo* studies mentioned above have been carried out using mouse models [16, 23–28].

## 4. *In Vivo* Studies on NET Formation

Extensive *in vitro* studies conducted on the formation of NETs and their relationship with diseases have raised many questions about their role *in vivo*. *In vivo* research on NETs is rendered complicated due to their fragility. However, some studies involve the use of complex methods to visualize NETs and determine their roles *in vivo*. Here, data from some promising *in vivo* studies, which have been published previously, has been summarized. Live cell imaging is the method of choice to directly visualize the morphologies and behaviors of NETs. McDonald et al. monitored the recruitment of neutrophils to the liver sinusoids during bacterial sepsis and visualized NET formation using live cell imaging and dual laser multichannel spinning-disk microscopy, respectively [23]. To visualize NETs *in vivo*, a cell-impermeable DNA dye was used to stain the extracellular web-like DNA structure created by the neutrophils within the lumens of the sinusoids.

Dissolution of the dye following the administration of DNase confirmed that the observed structures were composed of DNA. Additionally, fluorescently labeled antibodies specific to the constituents of NETs, which included histones and neutrophil elastase, were intravenously infused to confirm that the web-like DNA structures were NETs. McDonald et al. have demonstrated that NETs could form a large extracellular web within the liver sinusoids even under flow conditions. However, it was observed that they were not attached stably to the walls of the vessels of venules with the larger and higher shear compared to those of the slow-flowing liver sinusoids. A research by McDonald et al. revealed that the interaction between NETs and bacteria could be monitored *in vivo* upon infusion of fluorescently labeled *E. coli* into mice. Visualizing the formation of NETs *in vivo* has enabled us to confirm that they eliminate bacteria from dissemination [23].

Yipp et al. have developed assays to demonstrate the role of NETs *in vivo* using cell-impermeable/permeable DNA and fluorescently conjugated antibodies [16]. In the first assay, the formation of the extracellular web-like DNA structure was visualized *in vivo* using a cell-impermeable DNA dye infused 30 min prior to the administration of *S. aureus*. The second assay was designed using antibodies specific to the components of NETs such as histones and neutrophil elastase. Rapid release of histones and neutrophil elastase was found to occur in response to infection by *S. aureus* and *S. pyogenes* [16]. This result was contrary to that observed in the early *in vitro* study involving NETs, which revealed that neutrophils form NETs within hours of subjecting them to the inciting stimuli [5]. The third assay was designed using cell-permeable DNA and allowed direct visualization of the nucleus of intact neutrophils and quantification of the ratio of extracellular to intracellular DNA during sterile inflammation and infection [16]. Additionally, the results of the third assay helped determine the fate of the NET-releasing neutrophils. It was observed that NET-releasing neutrophils die following the NET release *in vitro* [5]. However, *in vivo* imaging of NETosis revealed that the NET-releasing neutrophils remain active and functional following the NET release [16].

Formation of NETs has also been monitored *in vivo* during sepsis. Widespread deposition of NETs was observed in murine livers upon subjecting them to sepsis induced by cecal ligation and puncture (CLP) [24]. NETs were observed *in vivo* through the intravascular administration of a cell-impermeable DNA dye and using fluorescently labeled antibodies against granular proteins [24]. It has been demonstrated that the intravascular injection of fluorescently labeled antibodies against the histone could stably and effectively stain NETs *in vivo* [23, 24]. A reduction in the amount of extracellular DNA was observed in mice subjected to CLP, which was stained using a cell-impermeable DNA dye in the liver and lung following the administration of an antibody for neutrophil depletion, DNase, and a neutrophil elastase inhibitor [24]. Interestingly, using a murine model, Cools-Lartigue et al. investigated the mechanism by which NETs interacted with tumor cells *in vivo* following their intravascular injection. They determined that tumor cells were trapped within the NETs present in the hepatic sinusoids of living mice and pulmonary capillaries *ex vivo* [24].

NETs have also been characterized *in vivo* in various organs of a murine sepsis model [27]. Tanaka et al. administrated a cell-impermeable DNA dye and anti-histone or anti-neutrophil elastase antibodies intravenously to GFP mice following lipopolysaccharide (LPS) stimulation to visualize the behavior of NETs in various organs. Firstly, different structures of NETs in the postcapillary venules of the cecum were observed, which were as follows: (1) reticular structures anchored to the leukocytes, (2) reticulolinear structures, (3) spotlike structures anchored to the leukocytes, (4) membranous structures on the surface of leukocytes, and (5) linear structures anchored to the leukocytes [27]. Surprisingly, the same study revealed that the occurrence of NETs *in vivo* is a rare event compared to the leukocyte-endothelial interaction [27]. Additionally, the presence of leukocytes was observed in the postcapillary venules of the cecum in mice treated with LPS, in which cytoplasmic vacuoles adhering to the vascular endothelium were found at the subcellular level. However, the occurrence of these characteristic leukocytes was less frequent in the arterioles and hepatic sinusoids [27]. Interestingly, few of these leukocytes were found to consist of cytoplasmic vacuoles, which released the NETs. It was unclear whether these cells were active or dead. However, the authors have stated that these cells might have undergone a suicidal form of NETosis. Secondly, the authors have reported a feature of NETs in the hepatic sinusoids of the liver, in which spotlike structures anchored to the leukocytes, cell-free DNA fragments, and those within platelet aggregates were observed in mice treated with LPS [27].

Tanaka et al. have reported the presence of few NETs in the hepatic sinusoids, an observation contrary to the previous findings of studies conducted *in vivo* on NETs by McDonald et al. [23, 24, 27]. Circulating cell-free NETs, which are characterized as fragmented or cotton-like structures, were detected in the blood of either hepatic sinusoids of the liver or postcapillary venules of the cecum following intraperitoneal administration of LPS [27]. It has been elucidated that the rate of blood flow might have played a role in determining the shape of cell-free NETs. Additionally, Tanaka et al. have reported that NETs could interact with the platelets, endothelial cells, or leukocyte-platelet aggregates *in vivo* [27]. This finding supports those revealed in previous reports on the interaction of NETs with platelets [29] and endothelial cells [30, 31]. However, further investigations are needed to determine the direct impact of NETs on these phenomena *in vivo*. However, this finding could be used to support those determined in the previous studies involving the interaction of NETs with platelets and endothelial cells [29].

Moreover, Kolaczkowska and the group determined that NETs could persist for up to 24 h in the liver vasculature after infection [25]. They revealed that the NETs are robust at 4 h and 9 h following infection with methicillin-resistant *S. aureus* (MRSA). Besides, the administration of DNase removes the extracellular DNA precisely but not the other NET components lining the walls of the liver sinusoids [25]. However, the presence of other components of NETs, such as histones and neutrophil elastase, might cause tissue injury even in the presence of a DNase inhibitor. Kolaczkowska et al. showed that NETs, not the pathogens, are entirely responsible for the liver damage inflicted after the infection with MRSA [25]. It has been determined that von Willebrand factor (VWF), which is normally associated with hemostasis and has been found to bind histones [32], could interact with NETs *in vivo* in the liver vasculature [25]. Adherence of histones and elastase to the sinusoidal endothelium and binding of the DNA to the vessel wall in mice pretreated with VWF-blocking antibodies could be prevented and reduced, respectively, under these conditions [25].

## 5. Conclusions

*In vivo* studies on NETosis have been conducted using advanced technology. These technologies have helped scientists understand the behavior of NETs at several sites in the animal models. The majority of the *in vivo* studies, if not all, have been conducted using mouse models, in which the behavior of NETs following the encounter with pathogens or tumor cells has been monitored. Although the use of mouse models has tremendously improved our understanding of the role of NETs *in vivo*, such studies have certain limitations. The *in vivo* studies of NETosis discussed in this review have involved the use of extensive surgical approaches in mice, followed by the administration of fluorescently labeled microorganisms and conjugated antibodies to generate infection and mark the NETs or neutrophils, respectively. These procedures might have impacted generated data, since they are invasive and do not take into account the fact that the animal is complete and intact. The use of other animal models such as zebrafish could help reduce the limitations of these studies. The zebrafish model is a powerful system used for the *in vivo* imaging of neutrophils because it complements the research conducted on mice and humans and also combines genetic modulation and *in vivo* imaging [33, 34]. Zebrafish has recently been accepted as a model system for the imaging of immune cells during wound healing and infection [35–38]. Imaging cell behaviors using a zebrafish model enables investigators to noninvasively examine the function of immune cells for an extended duration by regarding the whole animal as the study system [39]. The main advantage of using this model is that it enables researchers to fluorescently mark the transgenic lines within specific parts of the cells, which can consequently be visualized clearly during the early development of the zebrafish due to the optical transparency of its embryo [33, 39].
